# Dataset from proteomic analysis of rat, mouse, and human liver microsomes and S9 fractions

**DOI:** 10.1016/j.dib.2015.02.007

**Published:** 2015-02-24

**Authors:** Makan Golizeh, Christina Schneider, Leanne B. Ohlund, Lekha Sleno

**Affiliations:** Université du Québec à Montréal (UQÀM), Chemistry Department/Pharmaqam, Montréal, Que., Canada

## Abstract

Rat, mouse and human liver microsomes and S9 fractions were analyzed using an optimized method combining ion exchange fractionation of digested peptides, and ultra-high performance liquid chromatography (UHPLC) coupled to high resolution tandem mass spectrometry (HR-MS/MS). The mass spectrometry proteomics data have been deposited to the ProteomeXchange Consortium (http://proteomecentral.proteomexchange.org) via the PRIDE partner repository (Vizcaíno et al., 2013 [1]) with the dataset identifiers PXD000717, PXD000720, PXD000721, PXD000731, PXD000733 and PXD000734. Data related to the peptides (trypsin digests only) were also uploaded to Peptide Atlas (Farrah et al., 2013 [2]) and are available with the dataset identifiers PASS00407, PASS00409, PASS00411, PASS00412, PASS00413 and PASS00414. The present dataset is associated with a research article published in EuPA Open Proteomics [3].

**Specifications table**

**Table:** 

Subject area	*Chemistry, biology*
More specific subject area	*Proteomic analysis*
Type of data	*a)* *Raw and processed mass spectrometry data acquired by 2D-LC–MS/MS analysis of rat, mouse, and human liver microsomes and S9 fractions* *b)* *Excel datasheets with identified proteins and corresponding peptides from each analyzed sample*
How data was acquired	*2D-LC–MS/MS using Agilent 1200 HPLC, Shimadzu Nexera UHPLC, and AB Sciex TripleTOF 5600 mass spectrometer*
Data format	*.wiff,.wiff.scan (raw files)*
	.group,.xml (search files)
	*.mgf (peak files)*
	*.xlsx (false-discovery rate analysis and processed results)*
Experimental factors	*No sample pretreatment applied*
Experimental features	*Liver microsomes/S9 fractions were solubilized, denatured, and subjected to a trypsin/pepsin parallel dual-digestion. Digested peptides were fractionated using strong cation exchange (SCX) chromatography prior to UHPLC–MS/MS with subsequent data-mining and bioinformatics analysis.*
Data source location	Université du Québec à Montréal (UQÀM), Chemistry Department, Montréal, Que., Canada
Data accessibility	The mass spectrometry proteomics data have been deposited to the ProteomeXchange Consortium via the PRIDE partner repository [1] with the dataset identifiers PXD000717, PXD000720, PXD000721, PXD000731, PXD000733 and PXD000734. Data related to the peptides (trypsin digests only) were also uploaded to Peptide Atlas [2] and are available with the dataset identifiers PASS00407, PASS00409, PASS00411, PASS00412, PASS00413 and PASS00414.

**Value of the data**

• Liver proteins identified in microsomal and S9 fractions with high sequence coverage.
• Comprehensive list of proteotypic peptides reported for each identified protein enables more targeted analyses.
• Cross-species analysis of rat, mouse and human liver microsomes and S9 fractions.
• Multiple-sequence alignment (MSA) analysis possible between proteins exclusively found in each species.

## 1 Experimental design, materials and methods

Rat, mouse, and human liver microsomes or S9 fractions (0.6 mg protein, *n*=2) were solubilized in 2% SDS solution (1:1 v/v ratio) and then diluted with 0.1 M ammonium bicarbonate (pH 8.5) prior to reductive alkylation with dithiothreitol (2.5 mM) and iodoacetamide (5 mM). Additional ammonium bicarbonate (for trypsin), or 0.2% trifluoroacetic acid in 20% methanol (for pepsin), was added for an overnight digestion at a 1:50 (w/w) enzyme/protein ratio. Digests were neutralized, diluted with water, and subjected to solid-phase extraction (SPE) on a 1 cm^3^ (30 mg) OASIS HLB cartridge (Waters, Milford, MA), eluting with 100% methanol (1 ml). Eluates were evaporated to dryness under vacuum, reconstituted in SCX buffer A (see below), and injected (100 μl, 0.5 mg protein) onto a Zorbax 300-SCX 150×2.1 mm column with 5 μm (300 Å) particles (Agilent Technologies, Palo Alto, CA) using an Agilent 1200 series HPLC equipped with a binary pump, degasser, diode array detector and fraction collector. SCX fractionation was performed (250 μl/min) with a gradient of 0–50% B in 15 min, up to 100% B at 25 min, then held for an additional 5 min at 100% B, where buffers A and B were 10 mM potassium dihydrogen phosphate in 25% acetonitrile (pH 2.75), and 1 M potassium chloride in buffer A (pH 2.75), respectively. UV absorbance was monitored at 220 and 280 nm. For trypsin samples, 3 min (0.75 ml) fractions were aliquoted into 1.5 ml tubes between 1.5 and 19.5 min, while for pepsin, 4 min (1.0 ml) fractions were collected between 1.5 and 25.5 min. Fractions were evaporated to dryness under vacuum and kept at −30 °C.

Dried fractions were reconstituted in 10% acetonitrile (100 μl) and injected (20 μl) onto an Aeris PEPTIDE XB-C18 100×2.1 mm column, with solid core 1.7 μm particles (100 Å) (Phenomenex, Torrance, CA). RP-LC was performed (300 μl/min, 40 °C) on a Nexera UHPLC system (Shimadzu, Columbia, MD) with water (A) and acetonitrile (B), both containing 0.1% formic acid with a gradient of 5% B held for 2 min, increased linearly to reach 30% B at 24 min, to 50% B at 26 min, then to 85% B at 26.5 min and held for 2 min. MS and MS/MS spectra were collected on a high-resolution hybrid quadrupole-time-of-flight (QqTOF) TripleTOF 5600 mass spectrometer (AB Sciex, Concord, Ont.) equipped with a DuoSpray ion source in positive ion mode. The instrument performed a survey TOF-MS acquisition from *m*/*z* 140–1250 (250 ms accumulation time), followed by MS/MS on the 15 most intense precursor ions from *m*/*z* 250−1250 (precursors excluded for 20 s after two occurrences) using information-dependent acquisition (IDA) with dynamic background subtraction (DBS). Each MS/MS acquisition (*m*/*z* 80–1500) had an accumulation time of 50 ms and collision energy of 30±10 V. The total cycle time was 1.05 s.

MS/MS files were combined and searched against the UniProt protein database (www.uniprot.org, release date 26/06/2013) by ProteinPilot software (version 4.1) using Paragon algorithm [4] using a thorough ID search with no specified enzyme and carbamoylation as a fixed cysteine modification. The search was performed for +2 to +4 charge states and MS tolerance was 0.05 Da on precursor ions and 0.1 Da on fragment ions. All duplicates were first processed alone, then together and finally tryptic and peptic digest for each sample were co-processed to obtain the total number of proteins and peptides. Proteins were identified with a 1% global false discovery rate (FDR) using a target-decoy database search algorithm [5] in ProteinPilot Descriptive Statistics Template (version 3.001p) (www.absciex.com/PDST). ProteinPilot Protein Alignment Template (version 2.000p) was also used for replicate analysis. The list of UniProt accession numbers from identified proteins was uploaded to NCBI Batch Entrez (www.ncbi.nlm.nih.gov/sites/batchentrez) to obtain the batch FASTA file, which was subsequently submitted to ExPASy (www.expasy.org) for determination of isoelectric point and monoisotopic molecular weight, to Phobius (phobius.sbc.su.se) [6] for prediction of integral membrane proteins, and to GRAVY Calculator (www.gravy-calculator.de) to compute grand average of hydropathy (GRAVY) scores. Processed lists of the identified proteins in rat, mouse, and human liver microsomes and S9 fractions, as well as the corresponding proteotypic peptides can be found in Supplemental Tables S1 and S2 (see Tables 1 and 2 for details).

## Funding sources

Financial support for this study was provided by the Natural Sciences and Engineering Research Council of Canada (Discovery Grant no. 355933-2011).

## Figures and Tables

**Table 1:** Processed data from proteomic analysis of rat, mouse, and human liver microsomes and S9 fractions.

Spices	Sample	ID	Protein data location	Peptide data location
*Rattus norvegicus*	Liver microsomes	RLM	Supplemental Table **S1-1**	Supplemental Table **S2-1**
	Liver S9 fraction	RLS	Supplemental Table **S1-2**	Supplemental Table **S2-2**
*Mus musculus*	Liver microsomes	MLM	Supplemental Table **S1-3**	Supplemental Table **S2-3**
	Liver S9 fraction	MLS	Supplemental Table **S1-4**	Supplemental Table **S2-4**
*Homo sapiens*	Liver microsomes	HLM	Supplemental Table **S1-5**	Supplemental Table **S2-5**
	Liver S9 fraction	HLS	Supplemental Table **S1-6**	Supplemental Table **S2-6**

**Table 2:** Dataset identifiers of the mass spectrometry data obtained from the analysis of rat, mouse, and human liver microsomes and S9 fractions on the public proteomics repositories.

Spices	Sample	ID	Protein dataset identifier (PRIDE)	Peptide dataset identifier (PeptideAtlas)
*Rattus norvegicus*	Liver microsomes	RLM	**PXD000720**	**PASS00407**
	Liver S9 fraction	RLS	**PXD000717**	**PASS00409**
*Mus musculus*	Liver microsomes	MLM	**PXD000731**	**PASS00411**
	Liver S9 fraction	MLS	**PXD000733**	**PASS00412**
*Homo sapiens*	Liver microsomes	HLM	**PXD000721**	**PASS00413**
	Liver S9 fraction	HLS	**PXD000734**	**PASS00414**

## Supporting information

Supporting Information. Table S1. Final list of the proteins identified in rat, mouse and human liver microsomes/S9 fractions at 1% FDR; Table S2. Final list of the prototypic peptides identified in rat, mouse and human liver microsomes/S9 fractions at 1% FDR.
